# InSilico DB genomic datasets hub: an efficient starting point for analyzing genome-wide studies in GenePattern, Integrative Genomics Viewer, and R/Bioconductor

**DOI:** 10.1186/gb-2012-13-11-r104

**Published:** 2012-11-18

**Authors:** Alain Coletta, Colin Molter, Robin Duqué, David Steenhoff, Jonatan Taminau, Virginie de Schaetzen, Stijn Meganck, Cosmin Lazar, David Venet, Vincent Detours, Ann Nowé, Hugues Bersini, David Y Weiss Solís

**Affiliations:** 1 Institut de Recherches Interdisciplinaires et de Developpements en Intelligence Artificielle, the Computer and Decision Engineering Department, Universite Libre de Bruxelles, 87 av. Adolphe Buyl, 1050 Bruxelles, Belgium; 2 IRIBHM, School of Medicine, Campus Hospitalo-Facultaire Erasme, Universite Libre de Bruxelles, 808 route de Lennik, B1070 Bruxelles, Belgium; 3 The Computational Modeling Lab (CoMo) Department of Computer Science Faculty of Sciences (WE) Vrije Universiteit Brussel, Pleinlaan 2, B-1050 Brussels, Belgium

## Abstract

Genomics datasets are increasingly useful for gaining biomedical insights, with adoption in the clinic underway. However, multiple hurdles related to data management stand in the way of their efficient large-scale utilization. The solution proposed is a web-based data storage hub. Having clear focus, flexibility and adaptability, InSilico DB seamlessly connects genomics dataset repositories to state-of-the-art and free GUI and command-line data analysis tools. The InSilico DB platform is a powerful collaborative environment, with advanced capabilities for biocuration, dataset sharing, and dataset subsetting and combination. InSilico DB is available from https://insilicodb.org.

## Rationale

Since the advent of microarrays and the recent adoption of next-generation sequencing (NGS) genome screening technologies, the usefulness of the resulting datasets for biomedical progress has been increasing. For example, these have been used for diagnosing individual tumors and discovering subclasses of disease previously undistinguishable by pathologists [1,2], paving the way towards personalized medicine.

As new knowledge and new perspectives are applied to published data, new insights are possible [3,4]. For example, indexes of differentiation in the thyroid can be derived from the reuse of public datasets [5], and general models of disease classification built [6]. Also, genome-wide data analysis methodologies can be tested comprehensively on a large scale [7]. Moreover, generic datasets are provided as resources with the purpose of being reused in the light of individual experiments, such as compendia of genome-wide responses to drug treatments [8], or of normal tissues, such as the Illumina Inc. Body Map [9]. These datasets are being used for biomedical applications such as drug repositioning [10], elucidation of cellular functional modules [11], cancer meta-analysis [12], the unraveling of biological factors underlying cancer survival [13], cancer diagnosis [14,15], and fundamental cancer research [16,17].

However, the complexity involved in managing these datasets makes the handling of the data and the reproducibility of research results very challenging [18-20]. InSilico DB aims to efficiently gather and distribute genomic datasets to unlock their potential. This is done by solving numerous issues around the data management that stand in the way of the efficient and rigorous utilization of this vast resource.

To start an analysis from available public data is difficult because the primary purpose of a repository is to guarantee the integrity of the data, not its usability. Indeed, prior to analysis, the raw data of genomic experiments is normalized or genome-aligned with sophisticated algorithms before being usable, the platform features are mapped to genes, and the meta-data (for example, patient annotations) are encoded in spreadsheet software and mapped to the individual experiments. Moreover, the normalization methods, the gene annotation, and the meta-data change in time and must be kept up-to-date. The meta-data can also be enriched with analysis results, such as disease classes newly defined by subgroup discovery. Finally, the data have to be transformed into the format accepted by the data analysis tools before it is ready for analysis. This process is tedious and notoriously error-prone (see, for example, [21]). InSilico DB makes this process automated and transparent to the user.

After the dataset is first published, it is desirable to preserve it for future use. This includes keeping track and properly indexing past experiments for efficient query to avoid unnecessary duplication of effort. Another important, and quite demanding, task is to obtain and annotate public datasets for comparison to newly generated datasets.

Adding a layer of complexity is the interdisciplinary nature of biomedical discovery, with bench biologists often preferring graphical user interface (GUI) analysis tools, such as GenePattern [22] or Integrative Genomics Viewer (IGV) [23], and biostatisticians requiring command-line programming environments such as R/Bioconductor [24]. The aforementioned platforms are tightly integrated into InSilico DB workflows, enabling collaborative discovery.

Some of these hurdles are accentuated with more voluminous NGS experiments. The transfer of the raw data generated through the internet is time-consuming, and personal computers are often not powerful enough to process the large amounts of data involved. InSilico DB proposes a solution to these issues by providing a web-based central warehouse containing ready-to-use genome-wide datasets. Detailed documentation and tutorials are available at the InSilico DB Genomic Datasets Hub.

## Overview of InSilico DB, browsing and searching content

**Figure 1:**
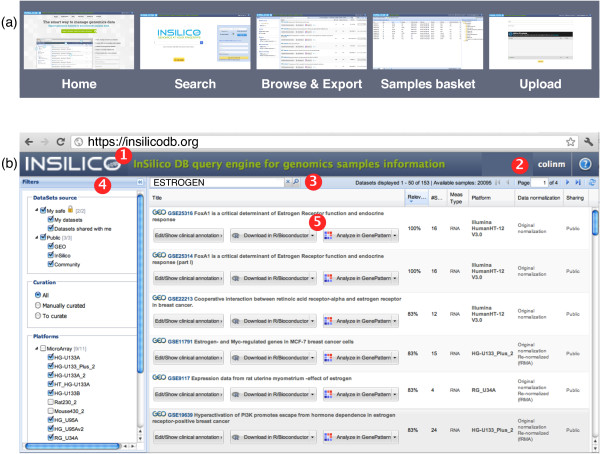
**Navigation and browse interface**. **(a) **Navigation pane, accessible at all times by clicking on the InSilico DB logo (see below). **(b) **The InSilico DB Browse & Export interface. The result after querying InSilico DB for the term 'Estrogen' is displayed. The main functions are indicated: 1, the InSilico DB logo is a link to access the navigation bar; 2, user information and feedback form; 3, search and find genomic datasets; 4, filter datasets, refine search results, manage and share sample collections; 5, results panel allowing the user to drill-down into information referring to desired datasets, and export it into supported analysis tools.

The InSilico DB Genomic Datasets Hub is populated with data imported from multiple sources; data can then be exported to multiple destinations in various ready-to-analyze formats. The main features of InSilico DB - search, browse, export and measurements grouping - are highlighted in Figure 1.

### Available public content

InSilico DB contains a large number of microarray and NGS datasets originating from public repositories, NCBI Gene Expression Omnibus (GEO) [25], Short Read Archive (SRA) [26], The Cancer Genome Atlas project (TCGA) [27] and the Broad Institute [28]. Currently, InSilico DB supports gene expression microarray Affymetrix and Illumina platforms, and Illumina NGS platforms (for an up-to-date list of available platforms, visit [29]). Clinical annotations associated with each sample are structured using the InSilico DB biocuration interface, a text-structuring tool that assists expert curators (see the 'Clinical annotations and biocuration' section below. As of August 2012, InSilico DB contains 6,784 public datasets accounting for 214,880 samples, among which 3,382 datasets and 151,131 samples have been manually curated. Owing to the accumulated in-house and contributed biocuration efforts, it is possible to accurately identify 78,953 tissue samples and 26,115 cell line samples. Table 1 gives more detailed statistics about the most commonly observed tissues.

**Table 1:** Total number of samples, cancer samples, and control samples for selected tissue types

Tissue type	Number of samples	Number of cancers	Number of controls
Breast	9,795	8,890	790
Bone marrow	7,594	5,113	2,019
Brain	7,424	1,214	1,886
Lung	4,823	2,636	1,085
Liver	3,781	875	436
Prostate	2,717	2,250	424
Colon	2,460	1,722	254
Blood	2,247	177	648
Kidney	2,116	913	348
Ovary	2,075	1,603	109
Skin	1 454	389	284
Lymph node	1,365	1,159	58

InSilico DB eases the accession of large, valuable datasets such as the Expression Project for Oncology (ExPO) [30], the Microarray Innovations in LEukemia (MILE) [31,32], the Connectivity Map (C-MAP and C-MAP 2.0) [8,33,34] or the Illumina Body Map 2.0 [9] datasets.

The entirety of the InSilico DB content provides a standard for stand-alone genome-wide analyses with standard software without the need of low-level data management related tasks.

### Browsing, filtering and searching InSilico DB content

Figure 1 illustrates the 'Browse & Export' interface. The interface is composed of two main panels: the center panel is a grid containing the results of the search; the left panel contains filters to fine-tune search results (Table 2 enumerates the available filters). Figure 1 shows the example of a query performed for the term 'Estrogen' resulting in the display of 153 datasets in the 'Browse & Export' interface. The user can then filter the results and sort them according to any column header - for example, the number of samples in the dataset. It is then possible for the user to drill-down on the samples information before selecting a dataset and exporting it to any of the supported analysis tools.

**Table 2:** Filters - InSilico web application

Filter	Function
**Dataset source**	Separates unpublished sample collections (such as obtained by grouping samples; My safe) from public-repository-derived content (Public). User-contributed datasets are only visible to the owner and his/her collaborators (see the 'A collaborative platform' section in the text). The Public filter can be expanded to select from the available public repositories. The InSilico filter contains InSilico DB compiled datasets. The Community filter contains datasets shared by InSilico DB users
	
**Curation**	Separates Manually curated datasets from To curate datasets. Manually curated clinical annotations have been structured and manually curated using the InSilico DB curation interface (see the 'Clinical annotations and biocuration' section in the text)
	
**Platforms**	Platforms are divided into two groups: gene expression microarray and next-generation sequencing. These groups can be expanded to select specific platforms
	
**Data pre-processing**	The data pre-processing filters are divided in microarray and next-generation sequencing groups. When raw data are available, InSilico DB pre-processes datasets using state-of-the-art algorithms, for example, fRMA for Affymetrix arrays, and Tophat-Cufflinks for RNA-Seq (see the 'Genomic dataset pre-processing pipelines' section in the text). The Original filter contains data as originally normalized submitted by the authors
	
**Measurement type**	The measurement type filters are divided into microarray (RNA) and next-generation sequencing (RNA-Seq, exome sequencing) groups

## Clinical annotations and biocuration

Online repositories of genomic datasets encourage the use of standards for describing the biological samples. For microarray datasets, the Minimum Information About a Microarray Experiment (MIAME) standard has been established [35]. This standard is particularly successful for describing experimental protocols. However, no standard has been accepted to describe biological samples information. As a consequence, clinical annotations are not standardized in the largest genomic datasets repository, GEO. A system that aims to structure the totality of the clinical information available would therefore necessitate a means of parsing free-form text.

InSilico DB proposes a bottom-up approach where users can structure samples meta-information, starting from unstructured annotations, and define their own structured vocabulary. Because the curation of a dataset may differ depending on the intended application - for example, smoking as a behavior or as a carcinogen - InSilico DB allows one dataset to have different curations. Additionally, InSilico DB accepts batch submissions from independent biocuration efforts. Batch submissions from the Broad Institute Library of Integrated Network-based Cellular Signature project [36] and from Gemma [37,38] have been received and added to InSilico DB.

**Figure 2:**
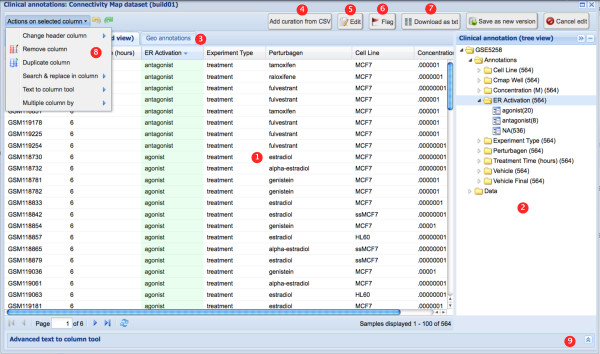
**Viewing and editing clinical annotations**. Clinical annotations of the C-MAP dataset. 1, spreadsheet view with headers representing clinical factors (for example, 'Perturbagen' or 'Cell Line'), and rows representing measurements and their associated values (for example, 'estradiol' or 'MCF7'). 2, Tree view condensed representation of the clinical annotations allowing fast identification of the number of samples annotated with a given clinical factor and clinical value (number in parentheses). 3, Central panel containing a second tab with additional meta-information. For GEO datasets, this second tab (named 'GEO annotations') links back to the original GEO web page dedicated to the dataset. 4, Curations upload button. 5, Edit curations. 6, Flag a curation - for example, in case of errors in the samples' annotations. 7, Download a curation. 8 and 9, After clicking on the 'Edit' button, the spreadsheet becomes fully editable and advanced tools ease the process of structuring annotations. Here, the curation of the C-MAP dataset is enriched with analysis results: the 'ER status' clinical factor is added with 'agonist' or 'antagonist' as values.

InSilico DB proposes an interface to visualize, curate and enrich clinical annotations of genomic datasets. Figure 2 shows the clinical annotations of the C-MAP dataset. Information is displayed using two alternative representations, a spreadsheet view and a tree view. In the spreadsheet view, headers represent clinical factors (for example, 'Cell Line' or 'Perturbagen'), and each row represents a measurement and its associated clinical values (for example, 'MCF7' or 'estradiol'). The tree view is a condensed representation of the clinical annotations that allows the user to quickly identify the number of samples annotated with given clinical factors and clinical values (the number of samples is indicated in parentheses).

Curations can be added from comma-separated value (CSV) files. Existing curations can be edited by using the curation interface, accessible through the 'Edit' button. To facilitate the curation of GEO studies, InSilico DB has imported all GEO curations and implemented a simple interface to assist the user in structuring this information.

**Figure 3:**
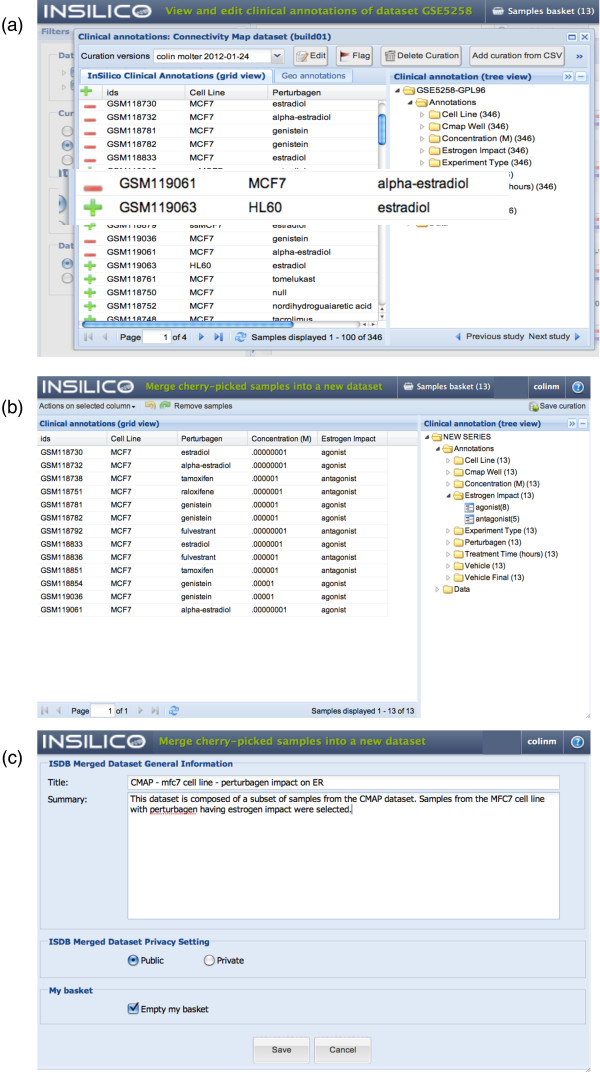
**Meta-dataset creation process**. **Process of grouping specific selected profiles**. **(a) **Press the green '+' (plus sign) toggle button to add samples to the 'Samples basket', and subsequently press the red '-' (minus sign) toggle button to remove samples. **(b) **In the samples basket, clinical annotations can be refined and standardized as explained in the 'Clinical annotations and biocuration' section. Additionally, samples can be removed using the 'tree view' actions. **(c) **To save, the user specifies a title, a summary and the desired privacy for the new dataset.

The curation process is based on the observation that the sample meta-data is amenable to a factor-value pair description, which can be represented in a tabular form (that is, columns correspond to factors and rows correspond to values). When the factor-value pairs are available in the standard GEO format, that is, factor-value pairs are separated by a comma character ',', and the factor is separated from the value by a colon character ':' (that is, 'key1:value1','key2:value2'), clicking on the 'guess' button of the 'Advanced text to column tool' will automatically perform the curation (this tool is shown collapsed at the bottom of the curation window shown in the bottom of Figure 3c; please refer to the online tutorials for a step-by-step video demonstration of this tool [39]). In case the information is not available in the standard GEO format, the user can proceed identically, except that she has to define her own separators to capture and structure the information into the final tabular form. We hope this collaborative tool will help the community structure all publicly available metadata in real-time as it gets published.

Additionally, the curation interface enables one to enrich existing curations to extend the set of factors describing a dataset. Specifically, in the spreadsheet view, each column header name in the meta-data table accessible through the curation interface corresponds to a factor describing the samples in a given dataset, and each cell under the column header is the value for that particular factor corresponding to the sample ID on a specific row of the table (Figure 3). Spreadsheet-like functionalities (accessible by clicking on the 'Actions on selected columns' button) allow users to, (i) edit the factor name, by editing the column header, (ii) remove factors by deleting a column, or (iii) add new factors by creating a new column or by duplicating an existing column.

A powerful application of this capability is to enrich the meta-data with analysis results. As an example, Figure 3 shows the process of enriching the existing C-MAP annotations with the results from Lamb *et al. *[8]. While studying the effect of estrogen receptor (ER) intracellular signaling pathway activation, the authors assessed the response of MCF7 cells to alpha-estradiol and beta-estradiol. They observed that the gene expression response of the cells was similar to independent experiments assessing the activation of the ER pathway (agonists, defined as a high 'connectivity score') and opposite from cells treated with fulverstrant, tamoxifen, and raloxifen acting as pathway inactivators (antagonists) [8]. In agreement, we added the 'er status' clinical factor and its corresponding clinical values, 'agonist' and 'antagonist', to the existing curation.

To ensure the traceability of the curation and reproducibility of the derived results, the curation version is uniquely identified and continuously available. The curation interface allows selection of a curation version, including the original curation, for example, from GEO (Figure 3a, top left corner. To assist the user in relating their curations to the original repository, the corresponding GEO web page is embedded in the side tab (the 'GEO annotations' tab in Figure 3a (right tab).

## Export

InSilico DB facilitates analysis by enabling a 'one-click export' of genomic datasets with curated clinical annotations to specific analysis platforms. Currently supported formats are R/Bioconductor [40], GenePattern [41] and IGV [42]. For microarray data, users can export molecular measurements per platform-specific probes or summarized by genes; and choose between the normalization provided by the original authors, or a normalization performed by InSilico DB using the fRMA R/Bioconductor package [43]. For RNA-Seq datasets, users can export gene-expression, splice junctions, transcript expression estimates, and differential expression results. For exome datasets, users can export annotated variants to IGV.

The InSilico DB content is also accessible from a programmatic interface that allows for batch queries through the R/Bioconductor package inSilicoDb [44].

To demonstrate how InSilico DB facilitates the access to genomic content, let us consider the following case. Suppose that a user wants to find genes correlated with ER pathway activation. After querying for the term 'estrogen' in InSilico DB, she selects three datasets for retrieval and analysis: (i) GSE20711 [45], a microarray dataset containing 87 samples from breast cancer patients with ER mutation status information (indicated as ER+ or ER-); (ii) GSE27003 [46], an RNA-Seq dataset with 8 samples from breast cancer-derived cell lines with ER+/ER- status; and (iii) ISDB6354, a subset of the C-MAP dataset containing the 13 MCF7 cell line samples that were treated with ER agonists or antagonists (the 'Grouping and sub-grouping' section explains how the subset is created).

**Figure 4:**
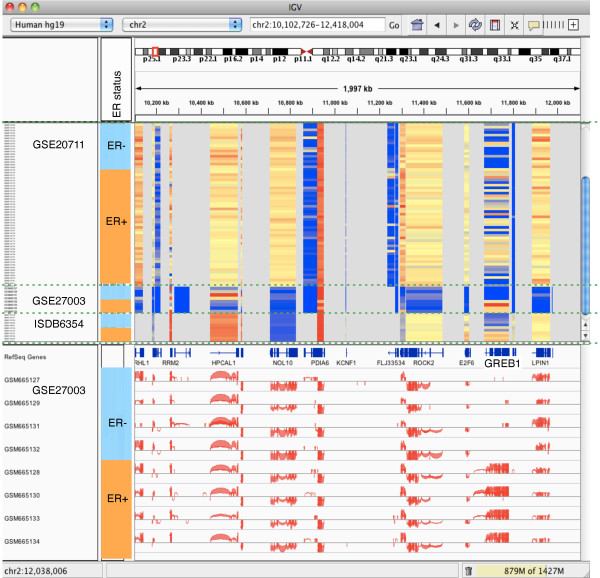
**Visualizing microarray and RNA-Seq gene expression data with IGV**. Joint visualization in IGV of three datasets: two microarray (GSE20711 and ISDB6354) and one RNA-Seq (GSE27003). The three datasets share the ER pathway activation status annotation (ER+/ER-). The top part of the central panel displays gene expression heatmaps. The bottom view displays the splice variants for the RNA-Seq dataset. The view is zoomed in on the locus containing an example gene, *GREB1*, in chromosome 2 that is regulated in the two microarray datasets.

For visualization and analysis, the user can export the data to GenePattern, or to her personal computer. Recently, GenomeSpace support has been implemented (see the 'Future directions' section below). Once in GenePattern or in the user's personal computer the data can be visualized using IGV. Figure 4 shows an example of visualization of these three datasets using IGV where expression data from the two microarray datasets can be examined simultaneously with expression data and splicing junctions from the RNA-Seq dataset [47]. She can then determine the genes with the most statistically different expression in the ER+/ER-phenotype using the ClassNeighbors GenePattern module [48]. Alternatively, she can retrieve the data from InSilico DB in R/Bioconductor format by executing the following code in an R console: library('inSilicoDb'); breastcancer = getDataset(gse='GSE20711',gpl='GPL570',norm='FRMA', genes=TRUE) rnaseq = getDataset(gse='GSE27003',gpl='GPL9115',norm='GENEEXPRESSION', genes=TRUE) cmap = getDataset(gse='ISDB6354',gpl='GPL96',norm='FRMA', genes=TRUE).

**Figure 5:**
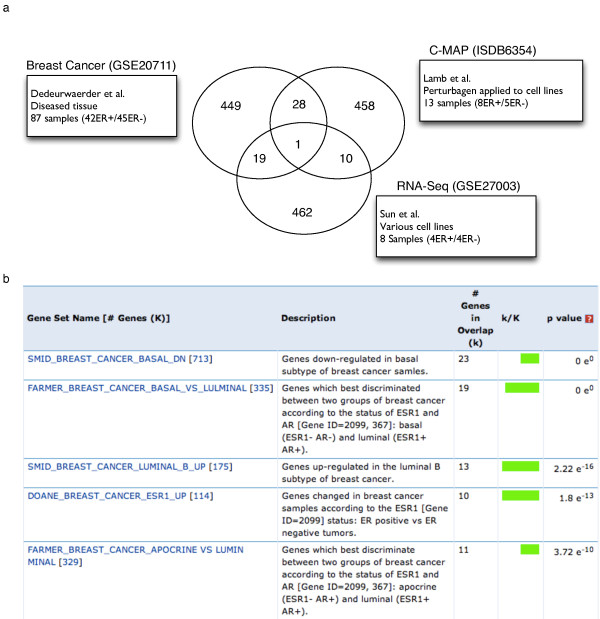
**Comparing signatures from differentially expressed genes between ER+/ER- samples in three conditions**. **(a) **Venn diagram showing the intersections of the signatures of the top 500 differentially expressed genes related to the ER pathway activation status for two microarray datasets (GSE20711 from Dedeurwaerder et al. [45], and ISDB6354 from Lamb *et al. *[8]) and one RNA-Seq dataset (GSE27003 from Sun *et al. *[46]) (for a full list of intersecting genes, see Additional file 3). **(b) **Top 5 curated gene sets associated with the 64 genes at the intersections obtained from MSigDB.

Once loaded into R/Bioconductor, the ER+/ER- samples' annotations are used to compute the top differentially expressed genes using the Limma package [49]. For the RNA-Seq dataset, differentially expressed genes are computed using the R/Bioconductor cummeRbund package [50]. Figure 5a shows a Venn diagram that illustrates the intersection of the computed differentially expressed genes (see [51] for details). Comparing the 58 intersecting genes to the Molecular Signatures Database (MSigDB) online collection of curated gene lists through the MSigDB web application [52] returns a list of highly significant ER-regulated pathways (Figure 5b).

## Grouping and sub-grouping

Large-scale meta-analyses, containing thousands of samples originating from various datasets from the public domain, have shed light on the structure of the gene 'expression space' [6,53,54]. Analyses that group phenotype-specific datasets have been successful in revealing novel gene signatures [55]. Selecting samples from large reference datasets and grouping them into meta-datasets can be challenging. For example, extracting the 33 thyroid cancer samples available from the ExPO dataset starting from the GEO repository would require one to download, process, curate and normalize either i) each sample separately, repeating the process 33 times, then reassembling them into a single dataset, or ii) the whole, very large (13.5 GB) dataset at once and then subsetting the resulting 33 out of 2,158 samples. To bypass this tedious and resource-hungry process, InSilico DB allows the user to select and group specific 'cherry picked' samples from one dataset, or even among various datasets. To select a sample, the user can click on the green plus sign appearing to the left of unselected samples in the curation view and, conversely, to de-select a sample, the user can click on the red minus sign appearing to the left of selected samples (Figure 3a). After all the desired samples have been selected, the user can view the selected sample collection by clicking 'Samples basket' (Figure 5b). The user can then (i) input a title and a description for the sample collection, (ii) set the permission to either keep the sample collection private or to share it with the community, and (iii) save it (Figure 5c). From then on, the newly formed sample collection is, for all purposes, a new dataset, but it belongs to the user who can access it by clicking on the 'My fafe' filter (Figure 1b, filter panel).

## A collaborative platform

InSilico DB is a collaborative platform that allows users to share genomic datasets. Dataset administrators can add/remove collaborators or groups of collaborators through a dedicated sharing interface. It is possible, as discussed in the 'Grouping and sub-grouping' section to create a new dataset by grouping samples from independent datasets. These newly generated datasets are private by default - that is, only the owner has access to them. Sharing preferences and the public status of the dataset can be changed by the owner. An owner of a dataset can make it public to the InSilico DB community or keep it private. A private dataset can be shared with collaborators who can be given read-only or read-and-write permissions. A user who has read-and-write permissions on a dataset can edit its sharing preferences. A tutorial to group and edit sharing permissions is available at [56]. It is also possible to share unpublished datasets with the community by contacting InSilico DB.

The support of GUI and command-line based interfaces to InSilico DB allows the collaboration of computational and bench biologists. For example, a biomedical expert can curate a given dataset using the web interface and visualize its expression data in GenePattern and can then in turn share this dataset with a computational collaborator who can perform further analyses through the command line with R/Bioconductor.

## Comparing InSilico DB with other data hubs

InSilico DB aims to greatly facilitate the use and re-use of genomic information content. For this task, InSilico DB is designed as a web-based data hub where datasets can be easily inserted, maintained, annotated, pre-processed and exported to various analysis tools or to other data hubs.

To highlight InSilico DB's strengths and weaknesses as well as to suggest future directions of development, it is useful to contrast InSilico DB with other genomic data hubs. Currently, the more mature platforms that have been published are GEO and Gene Expression Atlas [57]. Both are web-based data hubs for genomic research and a primary goal of each platform is to enable the re-use of published datasets. Table 3 summarizes and compares the features of these three platforms.

**Table 3:** Comparing InSilico DB with other databases

InSilico DB functionality	Description	GEO comparison	Expression Atlas
**Data sources**			
Public repositories	Support main platforms of GEO, SRA and notable public datasets	All platforms supported, limited to submissions	Support for Array-Express
Grouping	New datasets created by subsetting and combining datasets		
			
**Biocuration**			
Collaborative	Commonalizing biocuration effort through collaborative platform		
Vocabulary	User-defined tabular structured text	Free text	Ontologies & free text
Versioning	Multiple, user-contributed, batch submissions from biocuration projects, and in-house biocurator	In-house biocuration	In-house biocuration
Augment	Enrich curation with analysis results		
			
**Data management**			
	Processing raw data with latest algorithms, for example, fRMA for microarrays, TopHat+Cufflinks for RNA-Seq		
	Datasets can be shared at progressive levels, including groups, and published to the InSilico DB public repository		
			
**Data export**			
Raw data	Raw data are available for download	Raw data are available for download	Through ArrayExpress links
Output Format	Multiple ready-to-use analysis-tool-compatible format (GenePattern, R/Bioconductor and IGV compatible)	GEO-defined SOFT and text formats	
Programmatic access	InSilico Db R/Bioconductor package	Through third-party GEOquery R/Bioconductor package	

SRA, Short Read Archive.

## Materials and methods

### Genomic dataset pre-processing pipelines

All genomic data inserted in InSilico DB are associated with a genomic platform and a measurement type. These values define the pipeline used to pre-process all samples. R/Bioconductor and Python libraries are used on the back-end for data processing. For microarray data, background correction, normalization, and summarization are performed by applying the frma function of the fRMA R/Bioconductor package with the default parameters. Detailed documentation on microarray gene expression pre-processing pipelines can be found online in the InSilico DB website (see below for the specific URLs). For RNA-Seq data, read alignment and transcripts, gene expression abundance, and differential gene expression are computed using the Tophat-Cufflinks and cummeRbund pipelines [58]. For exome data, InSilico DB uses the genome analysis toolkit (GATK) 'best practice variant detection method' pipeline [59].

### Algorithm versions and parameters

InSilico DB was designed to enable biologists to efficiently gather and distribute large-scale genomic datasets. InSilico DB offers normalized data with the latest versions of state-of-the-art algorithms. When new algorithm versions appear, their previous versions are replaced by the most up-to-date versions in the InSilico DB pipelines and the data are re-normalized. This process ensures biologists always have access to data generated with the latest stable algorithm versions. Additionally, InSilico DB is synchronized daily with GEO to ensure the latest datasets are available.

To facilitate reproducibility all parameters and algorithm versions necessary to recompute the pre-processed data from the raw files are stored in the downloaded and exported datasets. Detailed documentation on how to access versioning and parameter information for each pre-processing pipeline can be found in the corresponding pre-processing documentation: RNA-Seq normalization pipelines are described at [60]; microarray normalization pipelines are described at [61]; exome normalization pipelines are described at [62].

### Search

For searching, InSilico DB uses Sphinx [63], an open source full-text search server, to query dataset metadata: titles, summaries, contributors, titles and abstracts of associated publications, clinical annotations of samples, and curators (Additional file 1). The full-text search server provides relevance scores through a search quality index.

### Backbone

As mentioned in the section 'Overview of InSilico DB, browsing and searching content' above, InSilico DB contains more than 200,000 genomic profiles that have been pre-processed according to specific pipelines. Giving the fast evolution of the genomics field, its pipelines and dependencies (for example, frma batch vectors or the genome annotations), InSilico DB has developed an architecture to update and re-run pre-processing pipelines for all associated profiles. To facilitate the task of pre-processing a lot of data simultaneously with minimal or no manual intervention, InSilico DB uses a workflow system developed *in situ*. This system, called the 'backbone' of InSilico DB, handles all server jobs, launches them on clusters by relying on a queue mechanism and monitors them on a database. Thanks to this 'backbone', pre-processing can be done on-demand: if a user request is not available, data are automatically pre-processed. After job completion, users receive an email with a link for an automatic download/export of the requested data (see Additional file 1 for a detailed description of the internal setup of InSilico DB).

### Architecture

InSilico DB is hosted at Universite Libre de Bruxelles (Brussels, Belgium). It runs on a 20-node cluster with the Linux operating system and SunGrid Engine queuing system. One machine is a dedicated web-server running Apache, one machine is a dedicated MySQL server, and one machine acts as a network attached storage with capacity of 50 TB. The front-end is written in javascript using ExtJS and JQuery libraries, the back-end is implemented in Zend PHP. A schema of the database can be found in Additional file 2.

## Future directions

Although hundreds of thousands of samples are publicly available, and several powerful analysis software solutions exist [22,24], the research community is facing a chasm between these two resources. To address the accessibility issues, the InSilico DB data hub contributes to resolving this problem by providing a centralized platform for the scientific community interested in using and sharing genome-wide datasets. For NGS experiments measuring gene expression, that is, RNA-Seq, microarray data provide a means of comparing the results to lower resolution but much larger published microarray datasets. For direct genome measurement, such as exome sequencing or whole-genome sequencing, gene expression data can serve as a functional validation. A future goal of InSilico DB is to add support for more genomic data types, such as single nucleotide polymorphism arrays, whole-genome sequencing data, methylation arrays, and microRNA platforms.

The pragmatic bottom-up approach to structuring clinical information used by InSilico DB has already yielded one of the largest collections of expert-reviewed genome-wide dataset annotations. A further step would involve relating the vocabularies defined by individual biocurators, or biocurating efforts, to overarching, well-defined ontologies. This would allow for the implementation of powerful mechanisms of querying InSilico DB, making meta-analyses even easier. Fortunately, biomedical ontologies exist (for example, the Unified Medical Language System (UMLS) [64]), as well as more general, bioscience-oriented data-exchange formats that are currently in active development [65]. InSilico DB accepts datasets annotated according to any standard, including these, and will in the future include tools to aid in the compliance to these standards. Future work will focus on the development of tools to assist users in adhering to a particular ontology system, or in linking their internally defined vocabularies to community-accepted standards. Another challenge is the identifiability of the experimental subjects that calls for a secure means of storing the data, sharing it with approved researchers only, and keeping track of access to files [66]. In this respect, the InSilico DB centralized warehousing approach would provide for a neutral location where data exchange can occur. Future work will thus focus on implementing highly secure mechanisms of data exchange.

To extend the number of supported bioinformatics analysis tools, InSilico DB will publicly release a web API to allow programmatic access to InSilico DB from third party tools. A pre-release can be found at [67]. Finally, by its participation in the GenomeSpace project [68], InSilico DB is part of a larger community-driven effort to improve interoperability of bioinformatics software and ultimately the usefulness of genomic data. GenomeSpace provides a central location on the cloud for storage of genome-wide datasets as well as generic means for analysis tools to connect to these datasets. InSilico DB is the first member of the GenomeSpace ecosystem providing expert-reviewed, richly annotated content gathered from public repositories providing a means for the biological researcher to unlock the potential of this vast resource.

## Abbreviations

C-MAP: Connectivity Map; ER: estrogen receptor; ExPO: Expression Project for Oncology; GEO, Gene Expression Omnibus; GUI: graphical user interface; IGV: Integrative Genomics Viewer; MSigDB: Molecular Signatures Database; NGS: next-generation sequencing.

## Authors' contributions

AN, HB, VD and DW conceived the project. AC, CM, RD, DS and DW designed the software. AC, CM, JT, RD, DS, DV, SM, CL and DW developed the software. JT, SM, CL and CM carried out validation research. VDS curated the datasets. CM, AC, and DW wrote the paper. All authors read and approved the final manuscript.

## Supplementary Material

Additional file 3 **Intersections of differentially expressed genes in three breast cancer studies**. Click here for file

Additional file 1 **InSilico DB architecture overview**. Click here for file

Additional file 2 **InSilico DB database schema**. Click here for file
