# Supplementary figures and images for: InSilico DB genomic datasets hub: an efficient starting point for analyzing genome-wide studies in GenePattern, Integrative Genomics Viewer, and R/Bioconductor
Source: Genome Biol. 2012 Nov 18;13(11):R104. doi: 10.1186/gb-2012-13-11-r104 (PMC3580496; doi:10.1186/gb-2012-13-11-r104)

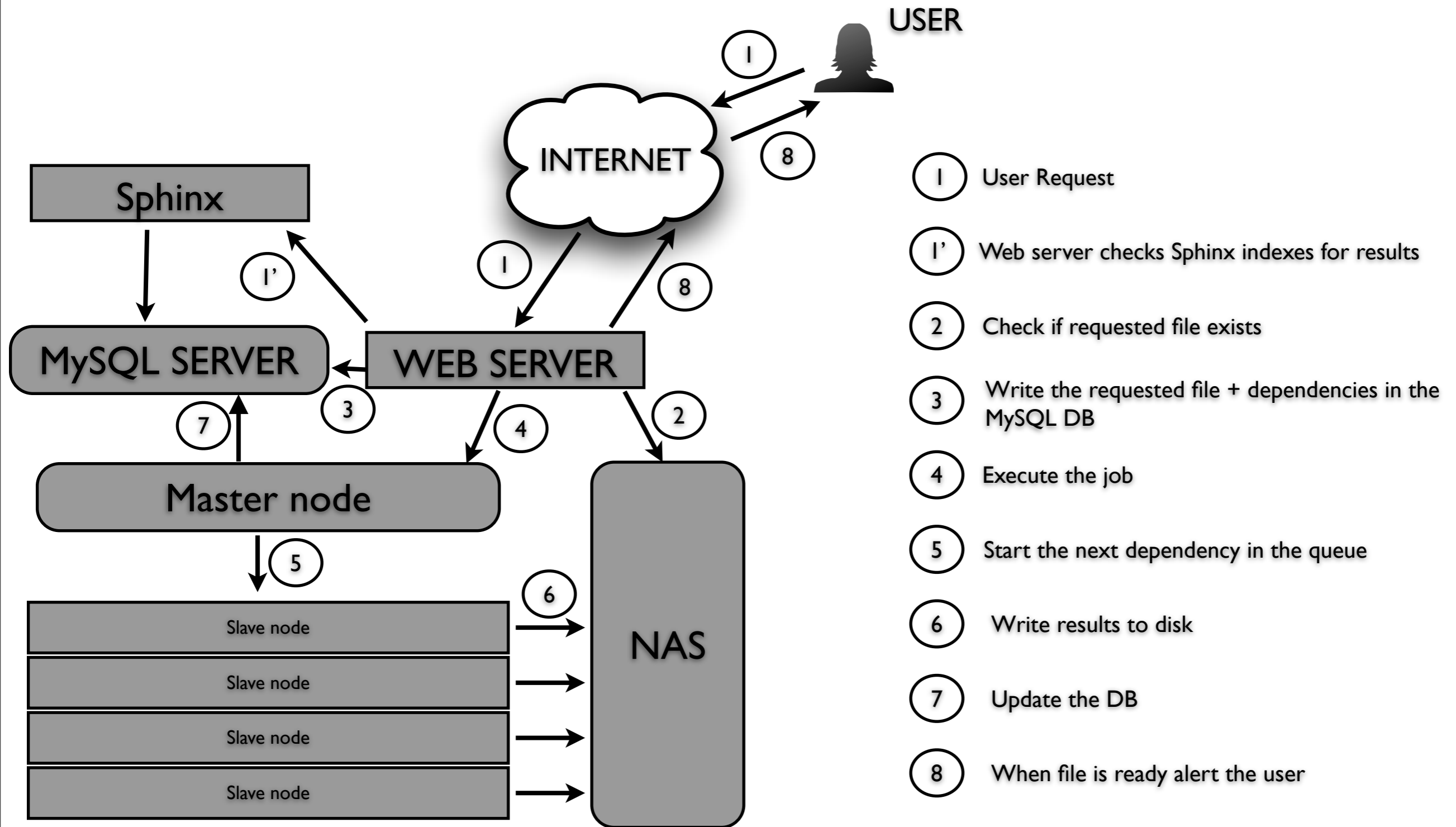

Supplement: Additional file 1 — InSilico DB architecture overview. [file gb-2012-13-11-r104-S1.PDF]

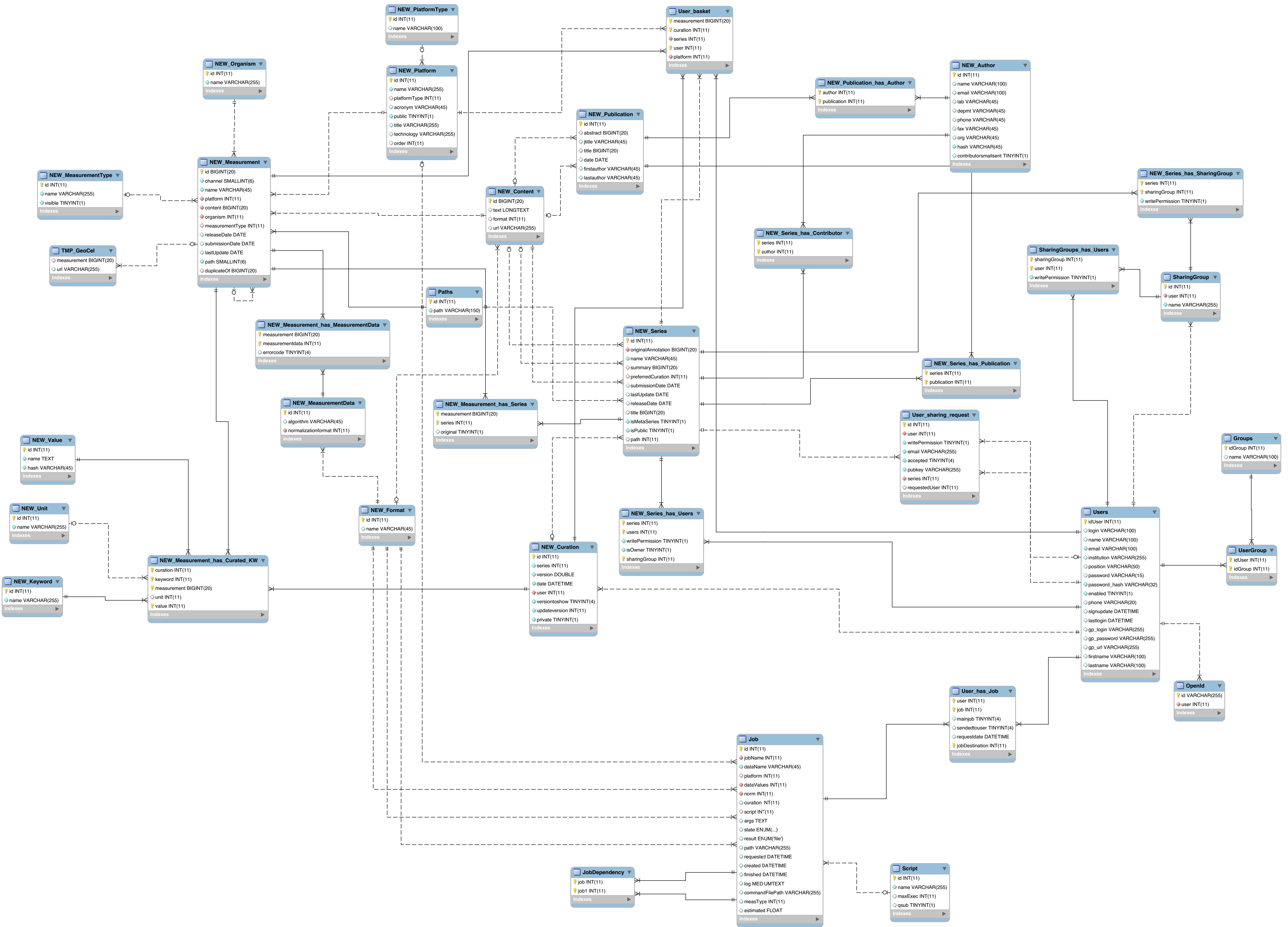

Supplement: Additional file 2 — InSilico DB database schema. [file gb-2012-13-11-r104-S2.PDF]
